# Enhancing Delirium Recognition: A 4AT Implementation Audit by the Mental Health Liaison Team at Southend Hospital

**DOI:** 10.1192/bjo.2025.10585

**Published:** 2025-06-20

**Authors:** Roxana Farcasan, Mohamed Ibrahim, Fiona McDowall

**Affiliations:** 1 Essex Partnership University NHS Foundation Trust, Rochford, United Kingdom; 2 Essex Partnership University NHS Foundation Trust, Southend, United Kingdom

## Abstract

**Aims:** This audit aimed to increase the use of the 4AT (4 A’s Test) within the Mental Health Liaison Team (MHLT) at Southend Hospital to enhance delirium awareness and recognition, thereby facilitating early discharge and improving patient outcomes. The 4AT is a validated and sensitive delirium detection tool, specifically designed for ease of clinical use, and supported by extensive diagnostic accuracy data from over 24 studies involving more than 5,000 observations. It is particularly useful as it can be administered to patients who are too sleepy or restless for traditional cognitive testing. Despite its advantages, compliance with 4AT documentation within the MHLT was initially low.

**Methods:** The audit was conducted in two cycles. The first cycle retrospectively reviewed patients aged 65 and over referred to the MHLT between 1 July 2023 and 30 September 2023. Out of 79 referrals, 66 patients were assessed, but only 5 (7.5%) had a documented 4AT score. To address this, interventions were implemented, including educational sessions for MHLT staff (doctors, nurses, and psychologists) on the use and benefits of the 4AT. Additionally, laminated 4AT tools were provided to staff as lanyard prompts to encourage use during assessments.

As aforementioned, not all referred patients were assessed by the MHLT. Some patients were discharged or, sadly, passed away prior to assessment, while others experienced worsening physical health conditions and were no longer fit for assessment. Additionally, some referrals only required medication advice, which did not warrant a full assessment.

**Results:** The second cycle evaluated data from 1 December 2023, to 29 February, 2024. Of 79 referrals, 64 patients were assessed, with 26 (40.6%) having a documented 4AT score, representing a nearly fivefold improvement in compliance. Despite this progress, further enhancements are needed to achieve consistent adherence.

**Conclusion:** The findings highlight the effectiveness of educational interventions and practical prompts in improving 4AT usage. Timely delirium identification is crucial for improving patient outcomes and facilitating early discharge, underscoring the importance of sustained efforts to integrate the 4AT into routine practice.

Recommendations include further staff education, integrating the 4AT into existing assessment forms, and conducting regular audits to ensure guideline adherence. This audit demonstrates the potential for improved delirium recognition and patient care through targeted interventions and ongoing monitoring. Future work can focus on expanding the use of the 4AT across the MHLT.

